# The transcription factor Jun is necessary for optic nerve regeneration in larval zebrafish

**DOI:** 10.1371/journal.pone.0313534

**Published:** 2025-03-10

**Authors:** Sarah C. Sarich, Virinchipuram S. Sreevidya, Ava J. Udvadia, Kurt R. Svoboda, Jennifer H. Gutzman

**Affiliations:** 1 Department of Biological Sciences, University of Wisconsin-Milwaukee, Milwaukee, Wisconsin, United States of America; 2 Joseph J. Zilber College of Public Health, University of Wisconsin-Milwaukee, Milwaukee, Wisconsin, United States of America; 3 Department of Biology, Appalachian State University, Boone, North Carolina, United States of America; Transilvania University of Brasov: Universitatea Transilvania din Brasov, ROMANIA

## Abstract

Damage to the axons of the adult mammalian central nervous system (CNS) from traumatic injury or neurodegenerative diseases often results in permanent loss of function due to failure of axons to regenerate. Zebrafish, however, can express regeneration-associated genes to revert CNS neurons to a growth-competent state and regenerate damaged axons to functionality. An established model for CNS axon regeneration is optic nerve injury in zebrafish, where it was previously shown that thousands of genes are temporally expressed during the regeneration time course. It is likely that hubs of key transcription factors, rather than individual factors regulate the temporal clusters of expression after injury to facilitate cell survival, regrowth, and synaptic targeting in the brain. One transcription factor of interest in orchestrating CNS axon regeneration is *jun*. However, it remains unclear if CNS regeneration can progress without Jun. To test this, a transgenic zebrafish line was developed to express a heat-shock inducible dominant negative Jun. Induction of dominant negative Jun downregulated endogenous *jun* expression and larvae with functional *jun* knockdown demonstrated impaired retinal ganglion cell axon regeneration. Analysis of select putative Jun target genes, previously shown to be upregulated in adult zebrafish optic nerve regeneration, demonstrated that with functional Jun knockdown, *atf3 and ascl1a* were significantly downregulated, and *sox11a* was upregulated at distinct time points. These results position *jun* as a key regulator for successful optic nerve regeneration, further distinguish the regeneration program from development, and advance our knowledge for the formation of future therapies to treat CNS damage.

## Introduction

Injury to axons of the mammalian central nervous system (CNS) from trauma or neurodegenerative diseases can result in permanent loss-of-function due to a failure in regeneration. The optic nerve, a component of the CNS, is comprised of retinal ganglion cell (RGC) axons that extend out of the eye, navigate to cross the optic chiasm, project through the optic tract, and form synapses with their appropriate targets in the brain. Damage to the optic nerve and optic neuropathies such as glaucoma, optic neuritis, and diabetic retinopathy lead to degeneration of the RGC axons, culminating in a loss of vision [1–4]. Current therapies only mitigate the progression of the diseases; therefore, it is necessary to identify key elements that will allow for functional regeneration of damaged RGC axons.

Mammalian embryonic RGCs are capable of regenerating damaged axons to their targets [5], but this capability is lost over time as the neurons mature [6,7]. Reverting neurons to a growth-competent state after injury requires the downregulation of genes associated with mature function and subsequent upregulation of growth-associated genes, many of which are expressed during development [7–10]. Regeneration also requires regrowing axons to overcome inhibitory signals present in the surrounding environment such as a glial scar, degenerating axon debris, and myelin debris [11]. Therefore, damaged neurons need to both induce appropriate regeneration-associated gene expression for a growth-competent state and overcome the inhibitory environment for functional regeneration.

Conversely, non-mammalian vertebrates such as zebrafish are capable of functional RGC axon regeneration [12]. Unlike mouse models, where 90% of RGCs die within 2–3 weeks after injury and survivors display limited growth [13–15], the majority of RGCs survive injury in zebrafish [16]. Minimal glial scaring occurs after injury and animals successfully revert their RGCs to a growth-competent state for axonal regrowth and synaptogenesis [17–19]. A previous study that analyzed gene expression during adult zebrafish optic nerve regeneration identified thousands of differentially expressed genes in temporal cascades across the regeneration time course [20]. It was concluded that rather than regulate thousands of transcripts individually, it is likely that a key cast of transcription factors orchestrate optic nerve regeneration, as transcription factors were differentially expressed over the time course and chromatin accessibility only significantly changed in the earliest and latest time points [20].

A particular transcription factor of interest in orchestrating CNS axon regeneration is *jun*. In the adult zebrafish study, *jun* was found to be upregulated early in response to injury, sustained over the course of regeneration, and downregulated upon synaptogenesis [20]. As a basic leucine zipper, Jun dimerizes with additional factors to form the AP-1 complex and regulate downstream gene expression [21]. *jun* was the only regeneration-associated transcription factor found to have accessible regulatory regions at the earliest timepoint after injury, leading to the hypothesis that it could play a key role in initiating a regenerative program [20]. Furthermore, *Jun* has long been associated with axon regeneration, having first been identified as upregulated in rat sciatic nerve injury [22], and found as essential for PNS axon regeneration [23,24].

Here, the zebrafish larval optic nerve transection model, established by Harvey et al. 2019 was used to understand the role of *jun* during RGC axon regeneration. *jun* expression was discovered to be downregulated in the developing retina by 48 hours post fertilization (hpf), but upon larval optic nerve transection, *jun* was rapidly induced and sustained, mirroring its expression pattern during adult zebrafish optic nerve regeneration [20]. It was further determined that *jun* is necessary for optic nerve regeneration by establishing independent transgenic lines of zebrafish with a heat-shock inducible dominant-negative Jun (DN-Jun) protein, capable of functional Jun knockdown. The efficacy of DN-Jun was validated and larvae with embryonic induction of DN-Jun displayed a variety of morphological defects. Induction of DN-Jun downregulated endogenous *jun* expression and, during optic nerve regeneration, larvae with functional *jun* knockdown demonstrated impaired RGC axon regeneration.

Finally, analysis of select putative Jun target genes, previously shown to have robust changes in expression during adult zebrafish optic nerve regeneration was conducted [20]. Genes that showed increased expression included *atf3, ascl1a,* and *sox11a;* whereas *e2f8, klf7b, nfil3,* and *stat5a* either had no change in expression or had a trend of downregulation over regeneration. With functional Jun knockdown, *atf3 and ascl1a* were significantly downregulated, and interestingly *sox11a* was upregulated at distinct time points. Together, these results demonstrate that RGCs are less likely to regenerate without functional *jun* expression and that putative Jun targets display varied expression patterns during larval regeneration with and without *jun*. These results support the hypothesis that *jun* is important for initiating and sustaining the signaling needed for functional optic nerve regeneration.

## Materials and methods

### Zebrafish husbandry and maintenance

This work was conducted by written consent in compliance with all regulations set forth by the University of Wisconsin-Milwaukee Institutional Animal Care and Use Committee (IACUC) at the University of Wisconsin-Milwaukee. The University of Wisconsin-Milwaukee is accredited by the American Association for Accreditation of Laboratory Animal Care (AAALAC). Zebrafish were raised according to the procedures by Westerfield [25]. Colonies were housed on recirculating racks (Aquaneering, San Diego, CA) at 28.5°C. Strains used in this study include wild-type (EkkWill, EK) and *Tg(isl2b:GFP)* [26]. Transgenic strains generated in the study include heat-shock inducible dominant negative Jun (DN-Jun) lines: *Tg(hsp70l:dnjun-2A-mCherry,Cryoaa:EGFP)*^*mke14Tg*^*, Tg(hsp70l:dnjun-2A-mCherry,Cryoaa:EGFP)*^*mke15Tg*^*, and Tg(hsp70l:dnjun-2A-mCherry,Cryoaa:EGFP)*^*mke17Tg*^*.* These lines were generated with a construct generously donated by Dr. Xianwen Peng [27] and will be referred to as *mke14Tg, mke15Tg, and mke17Tg* respectively.

### HSP70I:Jun-DN-mCherry transgenic line generation

The *Tg(hsp70l:dnjun-2A-mCherry,Cryoaa:EGFP)* plasmid was a gift sent from Dr. Xianweng Peng [27]. The plasmid was transformed and purified with Promega’s PureYield Plasmid Miniprep System (#A1222) according to manufacturer’s instructions. Confirmation of the plasmid was done via diagnostic double digest, using NcoI and AgeI (New England Biolabs). The product was run on a 1.1% agarose gel in TAE buffer at 100 V for 30 minutes. Resulting bands were the predicted lengths of 569 bp, 435 bp, and 7296 bp.

Using filament needles (World Precision Instruments 6 in Borosil GL w/fil 1.0 mm OD #1B100F-6) EK zebrafish were co-injected at the 1-cell stage with 100 ng/µL of the purified *Tg(hsp70l:dnjun-2A-mCherry,Cryoaa:EGFP)* construct and 100 ng/µL of Tol2 mRNA. Larvae were screened for EGFP expression in the lens at 3 days post fertilization (dpf). EGFP positive fish were raised and crossed with EK to identify founders. Three founders were identified based on correct expression and localization of EGFP in the lens and appropriate mCherry expression under heat shock and no heat shock conditions. These lines were named *Tg(hsp70l:dnjun-2A-mCherry,Cryoaa:EGFP)*^*mke14Tg*^*, (mke14Tg); Tg(hsp70l:dnjun-2A-mCherry,Cryoaa:EGFP)*^*mke15Tg*^*, (mke15Tg); and Tg(hsp70l:dnjun-2A-mCherry,Cryoaa:EGFP)*^*mke17Tg*^*, (mke17Tg)* (S1 Table).

For experiments utilizing the *mke14Tg*, *mke15Tg*, or *mke17Tg* lines, larvae were screened to confirm expression of EGFP in the lens, indicating the presence of the heat shock inducible DN-Jun transgene. Then, the transgene positive fish were further screened following heat shock. Animals that expressed EGFP in the lens and ubiquitous mCherry after heat shock were referred to as DN-Jun(+). Animals that expressed EGFP in the lens but had undetectable mCherry after heat shock were referred to as DN-Jun(-).

### Whole mount *in situ* hybridization

*In situ* hybridization probes were generated according to Thisse and Thisse, 2008 [28]. RNA was extracted from zebrafish whole embryos using the Qiagen RNeasy Plus Universal Mini Kit (#73404). The Quanta Biosciences qScript Flex cDNA Synthesis Kit (#95049-100) was used to reverse transcribe cDNA. Primers contained the T3 RNA polymerase promoter sequence to generate RNA probes and were designed against zebrafish *jun* (ENSDARG00000043531, GRCz11). Primer sequences are shown below. 1652 base pairs (bp) out of a total 1950 bp of the *jun* transcript were amplified to generate the probe. Touchdown PCR [29] was used to generate the *jun* probe cDNA template. The product was cleaned and concentrated with the Zymo DNA Clean & Concentrator-5 kit (#D4003S). DIG-labeled RNA probes were ethanol precipitated and stored in 4:1 ddH_2_O: RNA Later (Sigma, #R0901) at −80°C. *In situ* hybridizations were performed according to [28] with slight modifications. Zebrafish were raised in PTU-Danieau (0.2 mM 1-phenyl-2-thiourea (PTU; #P0237, TCI America) in 30% Danieau), then staged and fixed in 4% Paraformaldehyde (PFA, #T353-500, Fisher Scientific) 1X phosphate buffered saline (PBS) at designated time points overnight at 4°C. After probe hybridization, anti-digoxigenin-AP Fab fragments (Roche, #11093274910) were diluted 1:7500 in blocking buffer. Samples were washed in PBST before incubation with NTMT buffer (100 mM NaCl, 100mM Tris pH 9.5, 50 mM MgCl_2_, 0.1% Tween 20). In the dark, fish were transferred to BM Purple AP Substrate (Roche, #11442074001), covered, and incubated overnight at room temperature. The next day, fish were washed, and whole heads or just eyes were either dissected for imaging or immediately post fixed in 4% PFA for 30 minutes at room temperature. Fish were stored in PBST at 4°C. To image, fish were placed in 1.5% low-melt agarose in PBS on a slide and placed on top of a white sheet of plastic under an Olympus SZX12 microscope. Images were captured on an Omax camera [30,31] and processed in FIJI [32].

Primer sequences for the *jun in situ* probes were as follows:

Jun Antisense Probe Forward: 5’-CATTAACCCTCACTAAAGGGAAGAGAGTATACAGACCTCGCCAC-3’
Jun Antisense Probe Reverse: 5’-GTAGCTCAGAAGTCAGGCAG-3’
Jun Sense Probe Forward: 5’-AGAGTATACAGACCTCGCCAC-3’
Jun Sense Probe Reverse: 5’-CATTAACCCTCACTAAAGGGAAGGTAGCTCAGAAGTCAGGCAG-3’

### Developmental heat shock and defect analysis

Zebrafish *mke14Tg, mke15Tg, mke17Tg and EK* lines were used to evaluate the efficacy of the DN-Jun protein. Fish from each DN-Jun line (*mke14Tg, mke15Tg, mke17Tg*) were crossed to *Tg(isl2b:GFP)* and the progeny were used for heat shock experiments. Fish that were positive for the transgene (EGFP in the lens) but did not have detectable expression of mCherry, and, therefore low or no expression of the DN-Jun protein, were referred to as DN-Jun(-). Siblings that were positive for the transgene (EGFP in the lens) and did express mCherry after heat shock, and therefore did express the DN-Jun protein, were referred to as DN-Jun(+). To induce expression of DN-Jun in zebrafish embryos, 30% Danieau was prewarmed in a water bath to 38°C. Fish were heat shocked for 60 minutes at sphere stage (~4 hpf) and again at 28 hpf. No more than 30 embryos were placed in a 60 x 15 mm petri dish at a time. The PTU-Danieau was replaced with prewarmed 30% Danieau, petri dishes were immediately covered either with parafilm or plastic wrap and a rubber band and placed into the 38°C water bath. After 60 minutes, fish were removed from the water bath and placed in an incubator at 28.5°C. At 72 hpf, fish were anesthetized in 0.016% tricaine, defects were scored, and imaged. Defect scoring was blinded and replicated independently by three individuals. Images were taken with a Lumenera Infinity 1 camera mounted to a Nikon SMZ 1500 stereomicroscope. Head morphology that deviated from the characteristics described in Kimmel *et al.* 1995 at 72 hpf were considered defects [33]. Briefly, normal 72 hpf protruding-mouth larvae should display a head-trunk angle of 25° and have the palatoquadrate and Meckel’s cartilage of the jaw extend anteriorly past the eye such that the Meckel’s cartilage lifts toward the upper jaw in front of the eyes [33]. In addition, the area of the ceratobranchial arches posterior to the eye, and the morphology of the palatoquadrate cartilage were noted. Heads that were observed to be smaller in size or visually deviate from these traits were considered to have a defect.

### Larval optic nerve transection

Larval optic nerve transection was performed as previously described [34]. In brief, *Tg(isl2b:GFP)* fish were crossed to each other ((*Tg(isl2b:GFP) X Tg(isl2b:GFP))* or *Tg(isl2b:GFP)* fish were crossed to *Tg(hsp70l:dnjun-2A-mCherry,Cryoaa:EGFP)*^*mke15Tg*^ *(mke15Tg)* (from here on annotated as *Tg(isl2b:GFP) X Tg(mke15Tg)*). Embryos were incubated in 30% Danieau then transferred PTU-Danieau in the dark upon shield stage (~6 hpf) to prevent pigment formation. To induce expression of the DN-Jun protein in experimental animals, *Tg(isl2b:GFP) X Tg(mke15Tg)* progeny were heat shocked in 30% Danieau for 45 minutes at 38°C at 4 dpf then returned to PTU-Danieau after returning to 28.5°C. After 24 hours, *Tg(isl2b:GFP) X Tg(mke15Tg)* progeny were then heat shocked again at 5 dpf in 30% Danieau and returned to PTU-Danieau afterwards. After the second heat-shock (at 5 dpf of age) fish were anesthetized in 0.016% tricaine (MS-222; Argent Chemical Labs, WA) in PTU-Danieau and mounted dorsal side up in 2.5% low-melt agarose (#BP165-25, Fisher Scientific) containing PTU-Tricaine on a microscope slide. Using a sharpened tungsten needle (#10130-05, Fine Science Tools, Tip Diameter 0.001mm, Rod Diameter 0.125 mm) the midpoint of the left optic nerve (between the eye and the optic chiasm) was transected under a fluorescent Zeiss SteREO Discovery.V8 microscope. Larvae were removed from the agarose and allowed to recover in Ginzburg Fish Ringers [25], 111.23 M NaCl, 3.35 M KCl, 2.72 M CaCl_2_ ∙ 2H_2_O, 2.38 M NaHCO_3_) for 1 hour, then returned to PTU-Danieau. Transection efficiency was evaluated at 24 hours post transection (hpt). Fish with full transections were kept for further experiments.

### Imaging and scoring axon regeneration after optic nerve transection

Axon regeneration after optic nerve transection was evaluated in *Tg(isl2b:GFP) and Tg(isl2b:GFP) X Tg(mke15Tg)* progeny. To image the visual path, 2.5% low melt agarose in PTU-Danieau was dropped via Pasteur pipette onto a coverslip and allowed to set. Once solid, but not hard, wells were poked into the agarose using a Pasteur pipette. Zebrafish were anesthetized, placed into the agarose wells face down, and positioned with forceps. Images were taken with a Photometrics CoolSNAP ES camera mounted to a Fluorescent Nikon Eclipse TE2000-U Inverted microscope and processed in FIJI [32].

### Retina dissections and RNA extraction

Whole larval retinas were dissected, and RNA was extracted as previously described [35]. Briefly, fish were sacrificed one at a time in an overdose of MS-222 and immediately transferred to the lid of a 60 x 15 mm petri dish with ice cold Ginzburg fish ringers. Two forceps were used, a #55 for larger cuts and to pin the larvae down, and a #5SF (Fine Science Tools, #11252-00) to remove the lens and RPE from the retina. The embryo head was pinched at the base of the hindbrain and separated from the body. Using the #55 forceps, the head was held in place while the #5SF forceps were used to puncture the cornea and remove it from the retina along with the RPE. To remove any remaining fragments of RPE or the lens, the retina was gently pressed to the petri dish with the #55 forceps and rolled around. Using a Pasteur pipette with a pulled fine tip, retinas were transferred to a 0.6 mL Eppendorf tube in Ginzburg fish ringers on ice. After 10 retinas were collected, they were pulsed down in a centrifuge at 3000 x g for 30 seconds, the ringers were removed, and 300 µL of cold TRIzol was added. On average, dissection of 10 retinas took between 20–25 minutes. Retinas were vortexed in TRIzol for 1 minute at max speed to homogenize, then stored at −80°C until RNA extraction. To extract RNA, 30 retinas were pooled together and brought to a final volume of 1 mL TRIzol for RNA extraction with chloroform. To efficiently extract small amounts of RNA, samples were transferred to phase lock gel – heavy (PLG) tubes (QuantaBio, #2302830) and incubated at room temperature for 5 minutes to allow phase separation. RNA was eluted in 12 µL of ddH_2_O.

### RT-qPCR analysis

The QuantaBio qScript Flex cDNA Synthesis kit (#95049) was used to generate cDNA; 50 ng of total RNA was used from each 30-retina RNA sample. Fast, 3-step cycling for RT-qPCR was performed with the PerfeCTa SYBR Green Fast Mix (QuantaBio, #95072) on a Bio Rad CFX96 Real-Time System. *Glmp* expression was used for normalization as its expression does not significantly change over the course of regeneration (S1 Fig) [36]. Primers used for RT-qPCR are listed in Table 1. *jun* primers amplified within the transactivation domain to measure endogenous *jun* expression and to avoid measuring DN-Jun expression. The 2^-ΔΔCT^ method was used to determine relative quantification of gene expression [37].

**Table 1:** Primers used in RT-qPCR.

Primers Used in RT-qPCR			
Gene	Forward (5’ to 3’)	Reverse (5’ to 3’)	Source
*ascl1a-201*	TTGAGCGTTCGTAAA	GCTGAAGGACTGGATT	[38]
*atf3-201*	ATCACAAACACACGCGCCTA	TTTGTGAAGTCGTCCAGCGT	[39]
*e2f8-201*	TCGATTTGGATGGAGAGGAG	ACCACAGGAGGACTGGACAC	This study
*glmp-201*	ATTGGCTATTGGATCACAGGAT	TAAACAGGGTGCAAATGAAATG	[36]
*jun-201*	TGACACTGGCGATAACTTTAC	TAGACATAGAAGGCAAAGCG	This study
*klf7b-202*	ACTTCAGCTGGTGCACGACAC	TCAAGTCCTCGTCGCTGTCTCC	This study
*nfil3-201*	GGTTACTAGAGATAACCACTGAATTC	GGTTACTAGAGATAACCACTGAATTC	[40]
*sox11a-201*	ACTTCGCCTCCTCCGCGCAA	AGCCCAGGCTGCCCTCGCTA	[41]
*stat5a-202*	TCATCTCCAGCTGTCAGTGC	AGGTGGAGAGGTCACGACTG	This study

Genes are noted with transcript names from Ensembl GRCz11 (see S2 Table for full Ensembl GRCz11 Transcript ID numbers).

### Statistical analyses

Significance of target gene expression compared at regeneration timepoints between *Tg(isl2b:GFP)* and *Tg(isl2b:GFP) X Tg(mke15Tg)* progeny was analyzed in Microsoft Excel with Student’s T-Test (Fig 7). All other statistical analyses were performed in GraphPad Prism 5 Windows, GraphPad Software, Boston, Massachusetts USA, www.graphpad.com. Significance for head defects between DN-Jun strains and comparison of gene expression between *Tg(isl2b:GFP)* and *Tg(isl2b:GFP) X Tg(mke15Tg)* progeny were analyzed via Two-way ANOVA followed by Bonferroni multiple comparisons test (Figs 4 and 5 and Table 2). *jun* and Jun putative target gene expression in *Tg(isl2b:GFP)* fish was analyzed with One-way ANOVA followed by Tukey’s multiple comparisons test (Fig 3 and S3 Table). To analyze the significance of regeneration progress between in *Tg(isl2b:GFP) X Tg(mke15Tg)* DN-Jun(-) vs DN-Jun(+) progeny, Chi-square analysis was performed using GraphPad InStat 3 (Windows, GraphPad Software, Boston, Massachusetts USA) (Fig 6).

**Table 2:** Observed anterior phenotypes at 72 hpf in different strains with and without early embryonic heat shock.

Strain	% Head Defect (n)		Significance
	**No heat shock**	**Heat Shock**	
***Wild-Type***	**10%** *(n = 3/30)*	**9%** *(n = 5/58)*	–
	**No heat shock Transgene (-)**	**No heat shock Transgene (** ^+^ **)**	
*Tg(isl2b) X T*g(***mke15Tg)*** and *Tg(isl2b) X T*g(***mke17Tg)***	**11%** *(n = 4/36)*	**7%** *(n = 4/54)*	–
	**Heat Shock** **DN-Jun(-)**	**Heat ShockDN-Jun(** ^+^ **)**	
*Tg(isl2b) x Tg(* ***mke14Tg)***	**26%** *(n = 11/43)*	**76%** *(n = 109/143)*	^**^ P < 0.01
*Tg(isl2b) x Tg(* ***mke15Tg)***	**21%** *(n = 23/110)*	**76%** *(n = 78/102)*	^**^ P < 0.01
*Tg(isl2b) x Tg(* ***mke17Tg)***	**21%** *(n = 13/63)*	**78%** *(n = 51/65)*	^**^ P < 0.01

**Fig 1:**
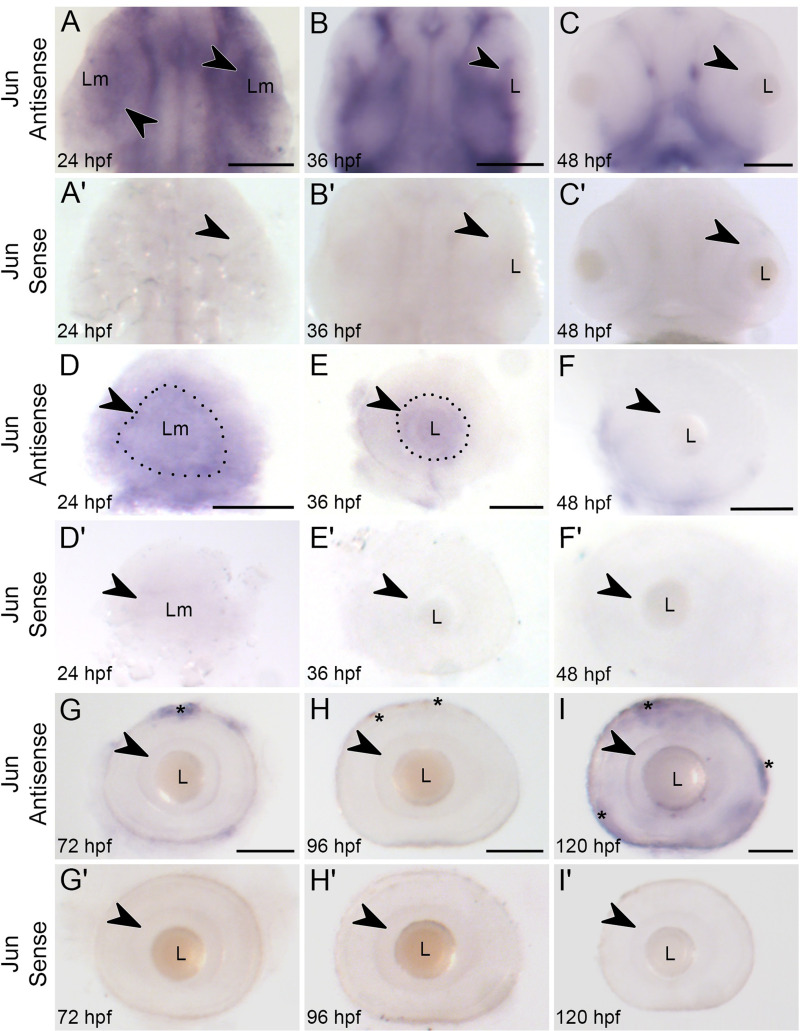
*Jun* expression in the developing retina is downregulated by 48 hpf. Whole mount *in situ* hybridization of *jun* expression in *Tg(isl2b:GFP)* embryos and larvae from 24 hpf – 120 hpf. (**A-C**) *jun* expression in the anterior region of the embryo at 24, 36, and 48 hpf, dorsal views. **A**. Arrowhead indicates *jun* expression in the optic lobe. **B**. Arrowhead indicates *jun* mRNA staining in the retinal progenitor cell layer. **C.** *jun* expression is absent in the ganglion cell layer, arrowhead. (**A’-C’**) *jun* sense controls in the anterior region. (**D-I’**) *jun* expression and sense controls in lateral views of dissected eyes from 24 hpf - 120 hpf. **D.** *jun* expression is present throughout the optic cup. A dotted line outlines the lens mass (Lm) and arrowhead indicates the most concentrated *jun* expression surrounding the lens mass. **E.** The retinal progenitor cell layer displays *jun* expression, outlined. This expression, arrowhead, cups the lens. (**F-I**) *jun* expression is undetectable in the ganglion cell layer at 48 hpf- 120 hpf. Arrowheads indicate the ganglion cell layer of the now formed retina. Asterisks indicate the outer pigmented epithelial layer. (**A-**C) Anterior is toward the top, **A-B**, dorsal view, **C**, ventral view. **A,D**, n = 44; **A’,D’** n = 6; **B,E,** n = 47; **B’,E’,** n = 5; **C,F,** n = 34; **C’,F’,** n = 4; **G,** n = 23; **G’,** n = 8; **H**, n = 26; **H’,** n = 4; **I,** n = 25; **I’,** n = 7. Lm =  lens mass; **L** =  lens. Scale bars =  100 µm.

**Fig 2:**
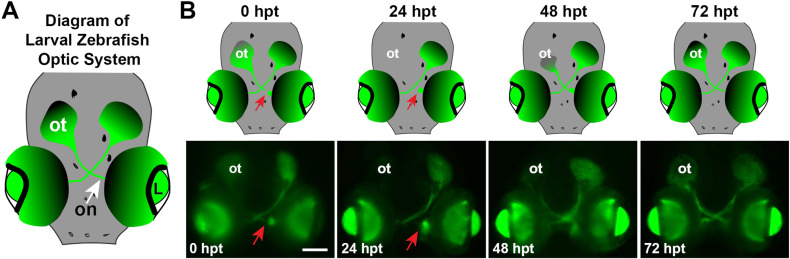
Larval zebrafish optic system, optic nerve transection, and axon regeneration timeline. **A.** Diagram of the uninjured 5 dpf zebrafish larvae optic system, dorsal view, anterior to the bottom. **B.** Diagrams and images of *Tg(isl2b:GFP)* larvae demonstrating the visual system, axonal injury, and regeneration process from 0 hpt - 72 hpt. Red arrows indicate points of optic nerve injury in the left eye. L, lens; ot, optic tectum; on, optic nerve. Scale bar =  100 µm.

**Fig 3:**
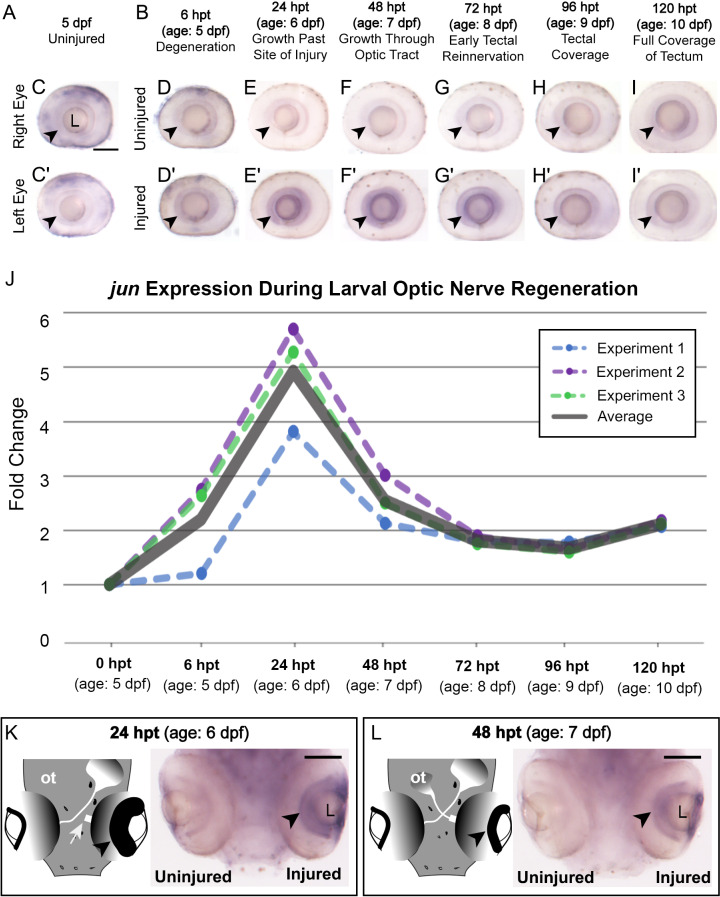
*Jun* mRNA expression is induced early upon optic nerve transection and RGC axon regeneration. (**A–**B) Labels describing stage for each dissected eye used for *in situ* hybridization experiments. (**C, C’**) *In situ* hybridization of *jun* in uninjured eyes at 5 dpf zebrafish. (**D–**I) *In situ* hybridization of *jun* in uninjured, right eye controls of larval zebrafish during optic nerve regeneration at each time point indicated. (**D’–I’**) *jun* expression in injured, left eyes during optic nerve regeneration at each time point indicated. (**C–I’**) Arrowheads indicate ganglion cell layer. 5 dpf uninjured n = 18; 6 hpt, n = 40; 24 hpt, n = 33; 48 hpt, n = 24; 72 hpt, n = 35; 96 hpt, n = 49;120 hpt, n = 49. **J.** RT-qPCR quantification of *jun* expression during optic nerve regeneration in the retina. The fold changes of *jun* during three separate replicates are plotted as dotted lines (green, blue, and purple), the average of the three replicates is plotted is shown as the thick, solid gray line. One-way ANOVA with Tukey’s Multiple Comparison post-test was used to evaluate all pairwise comparisons between time points and determine significance. As a result, fold change at 24 hpt (age: 6 dpf) was statistically significant compared to all other timepoints. 0 hpt, 6 hpt, 72 hpt, 96 hpt, 120 hpt, P < 0.001; 48 hpt, P < 0.01. **J.** Each time point for each replicate represents n = 30 pooled dissected retinas. (**K–**L) Diagrams of larvae showing post transection time points next to whole head *in situ* hybridization for *jun* comparing uninjured versus injury-induced *jun* expression in the same animal. White arrow indicates the site of transection. Arrowheads indicate ganglion cell layer. L, lens; ot, optic tectum;. Scale bar =  100 µm.

**Fig 4:**
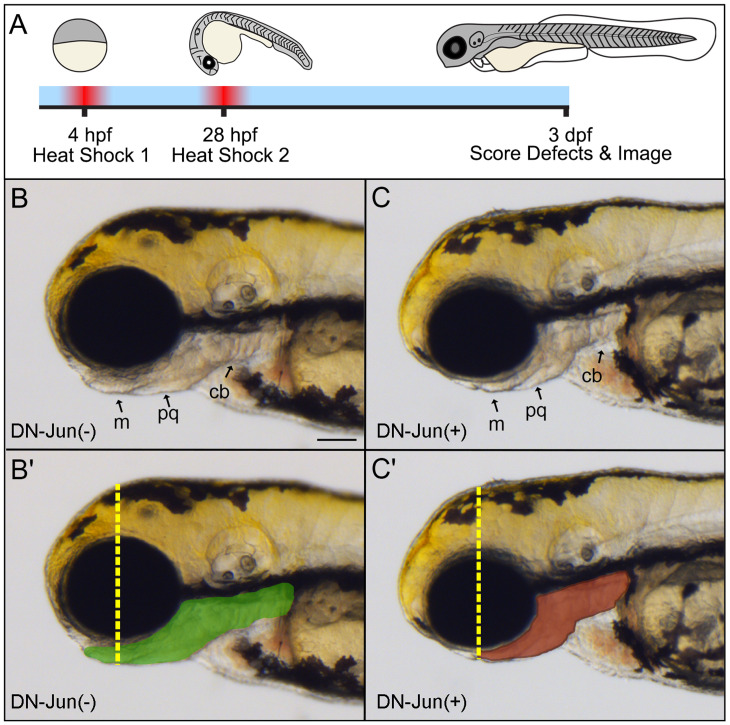
Early induction of DN-Jun results in developmental defects associated with *jun* expression. **A**. Graphical timeline of experimental workflow. Each DN-Jun strain (m*ke14Tg, mke15Tg, and mke17Tg)* was crossed with *Tg(isl2b:GFP)*; and progeny were heat shocked as described. Fish that were positive for the DN-Jun protein are referred to as DN-Jun(+), siblings that did not express DN-Jun are referred to as DN-Jun(-). (**B–C’**) Representative brightfield images following experimental procedure outlined in A. **B.** *Tg(isl2b:GFP) X Tg(mke17Tg)* DN-Jun(-) fish, 72 hpf, lateral view. **C**. *Tg(isl2b:GFP) X Tg(mke17Tg)* DN-Jun(+) fish, 72 hpf, lateral view. **B’**. Lower jaw and ceratobranchial arches outlined in green as region of interest, and yellow dotted line showing jaw extension in DN-Jun(-) fish. **C’**. Red outline of lower jaw and ceratobranchial arches as region of interest and yellow dotted line marking jaw extension past the central point of the eye in DN-Jun(+) fish. **m** =  Meckel’s cartilage, pq =  palatoquadrate cartilage, cb =  ceratobranchial arches. Scale bar =  100 µm.

**Fig 5:**
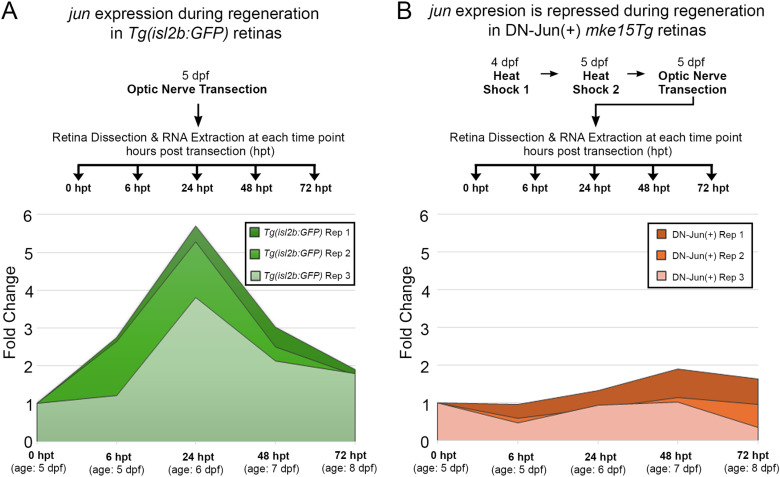
Induction of DN-Jun inhibits endogenous *jun* expression during larval optic nerve regeneration. **A.** Experimental workflow and RT-qPCR results for evaluation of *jun* expression during optic nerve regeneration in *Tg(isl2b:GFP)* fish. At 5 dpf, transections were performed on the left optic nerve. Retinas were dissected and RNA was extracted at 0 hpt, 6 hpt, 24 hpt, 48 hpt, and 72 hpt to use for RT-qPCR. **B.** Experimental workflow and RT-qPCR results for evaluation of *jun* expression during optic nerve regeneration in *Tg(isl2b:GFP) x Tg(mke15Tg)* DN-Jun(+) progeny. Larvae were heat shocked at 4 and 5 dpf, after which they were screened, and transections were performed on the left optic nerve. Retinas were dissected and RNA was extracted at 0 hpt, 6 hpt, 24 hpt, 48 hpt, and 72 hpt to use for RT-qPCR. Each time point for each replicate represents **n** =  30 pooled retinas. Two-way ANOVA with Bonferroni Multiple Comparisons was performed to determine statistical significance. ** =  p < 0.01; **** =  p < 0.0001.

**Fig 6:**
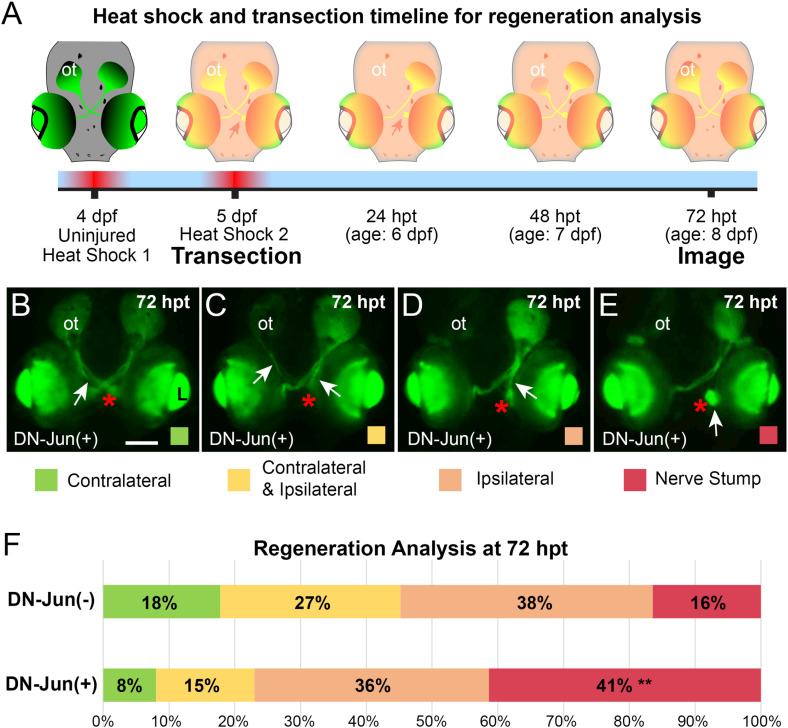
Induction of DN-Jun diminishes capacity for optic nerve regeneration. **A.** Experimental workflow. Left optic nerves of *Tg(isl2b:GFP)* X *Tg(mke15Tg)* progeny were transected after the second heat shock at 5 dpf then allowed to regenerate through 72 hpt (age: 8 dpf) when they were live imaged and analyzed. **(B–**E) Representative live fluorescent images of 72 hpt (age: 8 dpf) DN-Jun expressing larvae and the four potential regeneration outcomes after optic nerve transection. Red asterisks =  site of injury. **B.** Primarily contralateral axon regeneration, visible GFP labeled axons navigate to the opposite side’s optic tectum, white arrow. **C.** Contralateral and ipsilateral axon regrowth were both visible, and it was not possible to determine a dominant path, white arrows. **D.** Visible regenerating axons were primarily directed ipsilaterally, white arrow. **E.** No visible regeneration occurred; a nerve stump was present, white arrow. **F.** 100% stacked bar graph of regeneration progress in DN-Jun(-) and DN-Jun(+) fish. Color code indicates the primary axon growth trajectory identified. Green =  contralateral axon growth. Yellow =  both contralateral and ipsilateral axon growth. Orange =  ipsilateral axon growth. Red =  nerve stump, with no visible axon growth. Chi-square analysis was used to determine the significance between DN-Jun(-) and DN-Jun(+) regeneration phenotypes. The nerve stump phenotype was significantly increased in DN-Jun(+), ** **P** =  0.0025. Graph represents five biological replicates, DN-Jun(-) (negative for DN-Jun after heat shock) **n** =  73*;* DN-Jun(+) (positive for DN-Jun after heat shock) **n** =  87. ot =  optic tectum, **L** =  lens. Scale bar =  100 µm.

**Fig 7:**
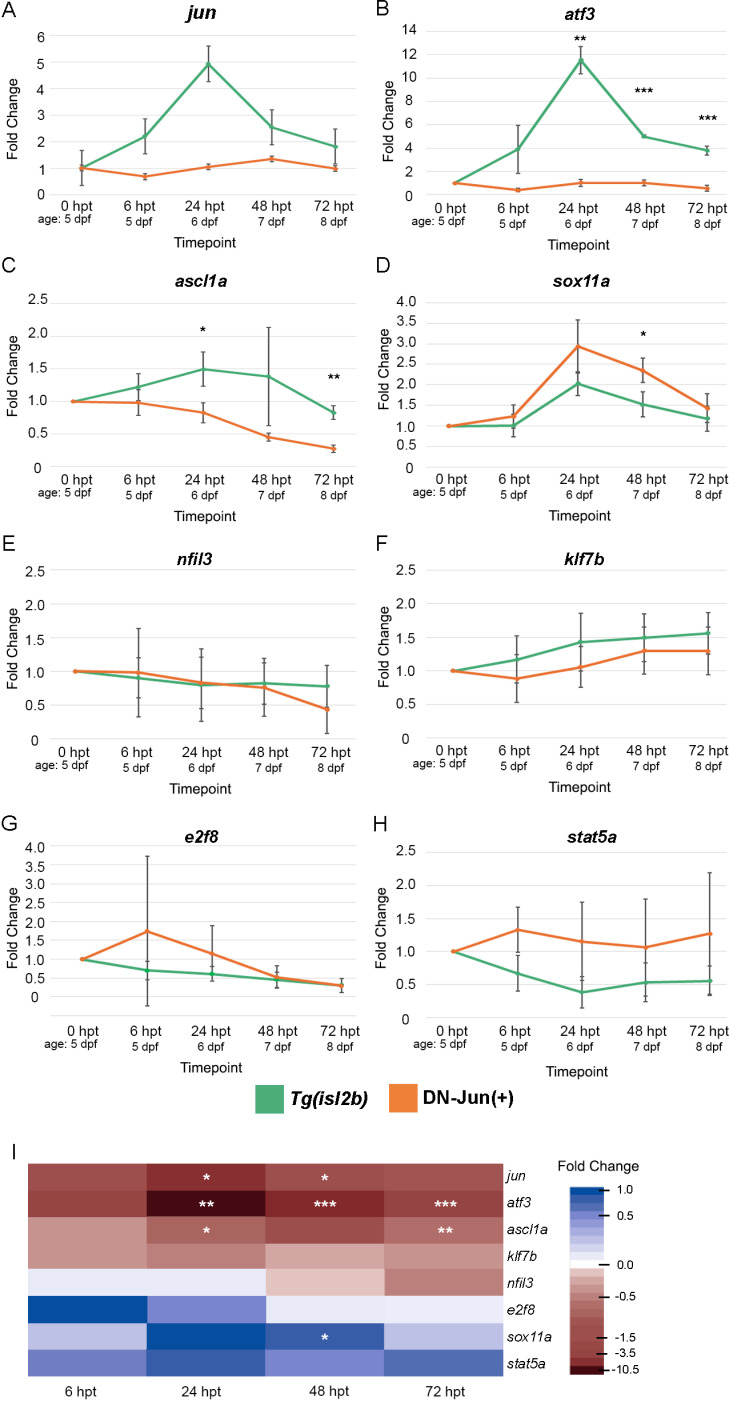
Putative Jun targets display varied expression patterns during larval optic nerve regeneration in control and Jun knockdown conditions. (**A–**H) RT-qPCR of *jun* and putative Jun target genes showing average expression during regeneration in *Tg(isl2b:GFP)* progeny (green lines) and *Tg(isl2b:GFP) X Tg(mke15Tg)* DN-Jun(+), Jun knockdown, progeny (orange lines). I: Heatmap showing difference in average fold change of *jun* and its putative targets’ expression between *Tg(isl2b:GFP)* and DN-Jun(+) conditions. **A–H.** Average fold change of three individual replicates were plotted for each condition and timepoint. Each timepoint required **n** =  90 retinas (30 retinas per replicate for 3 replicates) for both *Tg(isl2b:GFP)* and DN-Jun(+) experiments. Student’s T-Test was used to determine statistical significance between control and knockdown conditions for each timepoint. *  =  p < 0.05, ** =  p < 0.01, *** =  p < 0.001, **** =  p < 0.0001. All transcripts and primers for these data are shown in Table 1 and transcript ID can be found in S2 Table.

### Graphical figures

All graphical illustrations, timelines, and pathways are original illustrations created with Photoshop and Illustrator (Adobe).

## Results

Zebrafish retinal ganglion cells (RGCs) are the first to differentiate within the retina beginning at 27–28 hpf and continue for a few hours with the first post-mitotic cells being observed in the ventronasal quadrant [42–45]. Their axons begin to exit the eye at 30–32 hpf to form the optic nerve and by 34–36 hpf, the optic chiasm is formed [46]. In zebrafish, all RGC axons cross the optic chiasm towards the opposite, contralateral, side and the retinal RGC layer is established and distinguishable from the neuroepithelium by 36 hpf. By 48 hpf, RGC axons have begun to approach their major target, the optic tectum, and form synapses [42,45,47,48]. The retina continues to mature toward the periphery; by 50 hpf the inner plexiform layer, inner nuclear layer, outer plexiform layer, and outer nuclear layers are distinguishable [46]. By 60 hpf, RGC axon projections continue to branch and enter the tectal neuropil, and 90% of the retinal neurons are postmitotic [43]. At this stage, the first behavioral responses to light stimuli are active [49]. Finally, sophisticated visual behaviors such as the optokinetic response and prey capture begin at 96 hpf (4 days post fertilization, dpf) [50,51]. To understand the role of *jun* in orchestrating CNS axon regeneration during zebrafish larval optic nerve regeneration, it was first imperative to distinguish *jun* expression during retinal development from regeneration-associated *jun* expression. To accomplish this, whole mount *in situ* hybridization was performed on zebrafish embryos and larvae ranging from 24 hpf to 120 hpf (1–5 dpf) (Fig 1).

At 24 hpf, *jun* was expressed in a gradient medial to the lens mass of the optic cup (arrowhead, Fig 1A, D). At 36 hpf, the RGCs are surrounding the lens and projecting axons out of the eye to form the optic nerve and continued to express *jun* (Fig 1B, E). RGC axons reach the tectum by 48 hpf and no *jun* expression was detectable in the ganglion cell layer of the retina at this time (Fig 1C, F). The absence of *jun* mRNA in the RGCs persisted through 120 hpf, although pigment cells on the surface of the eyes displayed some *jun* expression (Fig 1G, H, I, asterisks). Some signal can also be seen between retinal layers; however, this signal is also visible in the sense control samples indicating that this is non-specific (Fig 1G–I’) [52]. These results demonstrate that *jun* is present in early differentiating retinal progenitor cells, and its expression persists as they mature and form axons to establish the optic nerve. Once the RGC axons reach the optic tectum and undergo synaptogenesis, *jun* is downregulated. This pattern of developmental *jun* expression in the RGCs indicates its timing of expression for appropriate development. Furthermore, the downregulation of *jun* expression in the developing eye beginning at 48 hpf can then be distinguished from future experiments where *jun* is upregulated.

*jun* has previously been shown to be upregulated in response to injury in the adult zebrafish optic nerve [20]. To confirm that expression of *jun* is upregulated in response to optic nerve transection in larvae, first larval optic nerve transections in 5 days post fertilization *Tg(isl2b:GFP)* fish were performed and the regeneration timeline was confirmed (Fig 2). A diagram defines the larval zebrafish optic system at the time when optic nerve transections are performed (Fig 2A). Then, regeneration time points are shown based on axonal positioning throughout the visual path as they degenerate following injury and then regenerate. As shown in diagrams and images of the *Tg(isl2b:GFP)* larvae (Fig 2B), immediately following optic nerve transection, the point of injury is visible (red arrow) and the optic tract and tectum are still labeled with GFP. By 24 hours post transection (hpt) (age: 6 dpf) the distal RGC axons within the contralateral tectum have degenerated, shown by an absence of fluorescence along the optic nerve and within the optic tectum. The point of injury is clearly visible (red arrow) and proximal axons are beginning to regrow from this point. After 48 hpt (age: 7 dpf), the axons have crossed the optic chiasm and have continued through where they are beginning to repopulate the optic tectum. Axons begin to cover the optic tectum by 72 hpt (age: 8 dpf) (Fig 2B), and will continue to cover the tectum over from 96 hpt (age: 9 dpf) to 120 hpt (age: 10 dpf) [34].

The next step to determine if expression of *jun* is upregulated in response to optic nerve transection in larvae, was to examine *jun* expression using *in situ* hybridization at distinct time points comparing uninjured and injured eyes (Fig 3A–B). Uninjured larvae did not show *jun* mRNA expression in the ganglion cell layer of either eye (arrowheads, Fig 3C–C’). For the transection experiments, the right eye served as an uninjured internal control (Fig 3D–I) and the left optic nerve was transected with a sharpened tungsten needle, labeled as the injured eye (Fig 3D’–I’). In the retina of transected optic nerves, *jun* was detectable in the RGCs as early as 6 hpt (age: 5 dpf), indicating its rapid upregulation in response to injury (arrowhead, Fig 3D–D’). By 24 hpt (age: 6 dpf), *jun* expression was abundant in the injured RGCs (arrowhead, Fig 3E–E’). *in situ* hybridization signal persisted through 48 hpt (age: 7 dpf) and 72 hpt (age: 8 dpf), as both time points continued to show staining of the RGCs (arrowheads, Fig 3F’–G’). As RGC axons cover the tectum at 96 hpt (age: 9 dpf), visualization of *jun* staining decreased in the ganglion cell layer (arrowhead, Fig 3H’). By 120 hpt (age: 10 dpf), there was no detectable *jun* mRNA staining in the RGCs, indicating its downregulation (arrowhead, Fig 3I’). At 120 hpt, non-specific signal was visible between retinal layers in both uninjured and injured eyes, similar to the developmental non-specific staining previously described (Fig 1).

To quantify these results at the molecular level, RT-qPCR was performed to measure *jun* expression in the retina during optic nerve regeneration using RNA extracted from dissected injured versus uninjured eyes (Fig 3J). Using RT-qPCR *jun* was found to be upregulated early at 6 hpt (age: 5 dpf), peaked at 24 hpt (age: 6 dpf), declined at 48 hpt (age: 7 dpf), then *jun* levels remained slightly above baseline from 72 – 120 hpt (age: 8 – 10 dpf) (Fig 3J). Whole head *in situ* hybridization animals were used to demonstrate the differences in expression between an injured and an uninjured eye within the same animal at the time points presented (Fig 3K–L). The pattern of *jun* expression generally resembled the staining observed in *in situ* hybridization experiments; however, given that *in situ* hybridization is not quantitative, a direct comparison of expression levels at each time point is only correlative.

*jun’s* expression pattern including early induction upon injury*,* persistent expression during regeneration, and downregulation upon reaching the optic tectum aligns with its expression pattern during optic nerve regeneration in adult zebrafish [20]. These results provide further evidence to support *jun* as an injury-inducible regeneration-associated gene (RAG) and distinguish its injury-induced expression from what was seen during retinal development. These experiments also further validate the larval optic nerve transection model for studying CNS regeneration-associated gene expression.

To test if RGC axon regeneration can progress without Jun, three independent heat-shock inducible dominant negative Jun (DN-Jun) transgenic lines (*mke14Tg, mke15Tg, mke17Tg)* were generated (see Materials and methods). Then each line was evaluated for the efficacy of DN-Jun protein function. It was hypothesized that induction of DN-Jun would outcompete and block endogenous Jun activity resulting in morphological defects. Phenotypes of the DN-Jun line have previously been characterized within the cardiac tissue [27] and *jun* mRNA is particularly concentrated in the zebrafish floor plate and developing ceratobranchial arches through 48 hpf (Fig 1A–C) [53]. Furthermore, c-Jun knockout mice die at E13 due to dysregulation of apoptosis, neural crest cell dysfunction, and abnormal hematopoiesis within the liver [54]. Therefore, it was expected that induced expression of DN-Jun would cause defects across the head and particularly within the ventral jaw regions due to the contribution of the neural crest to anterior jaw structures.

To test this, each of the DN-Jun strains (m*ke14Tg, mke15Tg, and mke17Tg)* were crossed with *Tg(isl2b:GFP)*. All embryos were heat shocked for 60 minutes at 38°C at shield stage, and heat shocked a second time 24 hours later at 28 hpf. Then, at 72 hpf (3 dpf) larvae were screened for EGFP expression in the lens to confirm the presence of the transgene. EFGP positive offspring were then screened for the heat shock-induced marker mCherry and categorized as offspring that were either DN-Jun positive (expressing DN-Jun protein after heat shock, DN-Jun(+)) or DN-Jun negative (not expressing DN-Jun protein after heat shock, DN-Jun(-)). The resultant phenotypes were evaluated (Fig 4 and Table 2). Normal 72 hpf larvae should display a head-trunk angle of 25° and have the palatoquadrate cartilage of the jaw extend anteriorly past the eye, allowing the Meckel’s cartilage to lift and form the lower jaw [33]. Heads that were observed to be smaller in size or visually deviated from these traits were considered to have a defect. At 72 hpf, only 9–10% of wild-type fish displayed defects, regardless of treatment with or without heat shock (Table 2). Similar results were found in progeny from *Tg(isl2b:GFP)* X *Tg(mke15Tg)* and *Tg(isl2b:GFP) X Tg(mke17Tg)* crosses in the absence of heat shock (Table 2).

Wild-type animals were treated with or without heat shock as outlined in Fig 4A and evaluated for anterior phenotypes at 72 hpf. Progeny from *Tg(isl2b:GFP)* X *Tg(mke15Tg)* and *Tg(isl2b:GFP) X Tg(mke17Tg)* crosses, in the absence of heat shock, were screened for the presence of the transgene (EFGP in the lens) and evaluated for anterior phenotypes at 72 hpf. The 3 independent DN-Jun expressing strains (*mke14Tg, mke15Tg, mke17Tg)* were crossed with *Tg(isl2b:GFP)* and their progeny were heat shocked as indicated in Fig 4A. DN-Jun(-) and DN-Jun(+) progeny were compared to wild-type embryos. Data from 3 replicates are shown, n’s represent fish with a head defect/total fish from all replicates. Two-way ANOVA with Bonferroni multiple comparisons post-hoc test was used to measure significance between DN-Jun(-) and DN-Jun(+) fish. ** =  P < 0.01.

Importantly, 76–78% of heat shock-induced DN-Jun(+) expressing fish displayed significant cranial developmental delays (Fig 4C and Table 2). DN-Jun(+) fish had increased head tail angles and smaller ceratobranchial arches, palatoquadrate cartilage, and Meckel’s cartilages that ultimately failed to lift and form the lower jaw. The overall size of the head and eye were diminished compared to heat shocked DN-Jun(-) animals, suggesting broad delays in developing cranial morphology. To clearly compare regions of ventral jaw cartilage between DN-Jun(-) and DN-Jun(+) fish, regions of interest were outlined in green (Fig 4B’, DN-Jun(-)) and red (Fig 4C’, DN-Jun(+)). The DN-Jun(+) fish clearly exhibit a smaller jaw area compared to DN-Jun(-) siblings, indicating that the DN-Jun protein successfully inhibited early endogenous Jun function within the developing ventral jaw cartilage.

Finally, while most heat shock-induced DN-Jun(-) fish exhibited appropriate development (Fig 4B and Table 2) there was a slightly higher percentage of head defects (21–26%) compared to heat shock wild-type animals and the non-heat shocked progeny with similar lines (7–11%) (Table 2). Given this difference, it is likely that the heat shock-induced DN-Jun(-) animals, although they had undetectable mCherry, were still induced to express enough DN-Jun to cause developmental defects in the head in a small percentage of embryos. There were no significant differences identified between DN-Jun strains. Therefore, the *mke15Tg* strain was used for the remainder of experiments.

*jun* regulatory regions contain *jun* DNA-binding motifs suggesting autoregulation [20,55]; therefore, it was further hypothesized that induction of DN-Jun would inhibit endogenous *jun* expression upregulation during optic nerve regeneration. To test this, the expression of *jun* during regeneration in DN-Jun(+) larvae was compared to *Tg(isl2b:GFP)* larvae, where *jun* expression peaked at 24 hours post transection during regeneration (Fig 5). *Tg(isl2b:GFP) X Tg(mke15Tg)* progeny were heat shocked twice to give the DN-Jun(+) protein sufficient time to be translated before optic nerve transection (Fig 5B). *Tg(isl2b:GFP)* larvae were used as controls for these experiments after it was confirmed that heat shock treatment did not induce *jun* expression at 24 hours post heat shock in the absence of transection. Further, the *Tg(isl2b:GFP)* transgenic line also served as a control to avoid effects from potential non-detectable DN-Jun expression after heat shock in DN-Jun(-) that could impact gene expression when analyzed by RT-qPCR (Table 2). The experimental workflow is shown for each condition (Fig 5). Results demonstrated that *jun* was significantly downregulated in DN-Jun(+) retinas at 6 and 24 hpt (age: 5 dpf and 6 dpf) (Fig 5B), indicating that DN-Jun successfully outcompeted endogenous Jun and inhibited its expression. Since *jun* induced expression was decreased by 48 and 72 hpt (age: 7 dpf – 8 dpf) in *Tg(isl2b:GFP)* animals, there were no significant differences in *jun* expression between *Tg(isl2b:GFP)*and DN-Jun(+) expressing fish at these time points.

Taken together, these results indicate that the heat shock inducible DN-Jun protein successfully inhibits endogenous *jun* expression induced by transection and that DN-Jun effectively prevents endogenous Jun from functioning properly during early embryonic cranial development (Fig 4). Not only does DN-Jun block Jun functionality, it also impedes *jun’s* autoregulation during optic nerve regeneration (Fig 5). This establishes the heat shock inducible DN-Jun zebrafish model as an effective way to manipulate *jun.*

After validating that induction of DN-Jun successfully inhibited endogenous Jun function during development and autoregulation of *jun* gene expression, the impact of DN-Jun on optic nerve regeneration was examined. It was hypothesized that optic nerve regeneration progress would be impaired following induction of DN-Jun. To test this, *Tg(isl2b:GFP)* X *Tg(mke15Tg)* progeny were heat shocked for 45 minutes at 38°C at 4 dpf and again at 5 dpf to ensure sufficient expression of DN-Jun prior to optic nerve transection. Optic nerve transections were performed after heat shock at 5 dpf and axon regrowth and guidance were evaluated at 72 hpt (age; 8 dpf) (Fig 6A). Larvae at 72 hpt (age: 8 dpf) displayed several different phenotypes that were categorized based on the visualization of GFP labeled axons and sorted into one of four general phenotypes: 1. RGC axon growth was primarily contralateral to the optic tectum, re-establishing the appearance of normal axon pattern during regeneration (Fig 6B, white arrow). 2. RGC axons were visible growing in both contralateral and ipsilateral directions during regeneration (Fig 6C, two white arrows). 3. RGC axons were primarily visible growing ipsilaterally during regeneration (Fig 6D, white arrow). 4. RGC axons were not visible during regeneration anywhere past the injury site, but still had surviving RGCs that developed a nerve stump (Fig 6E, white arrow).

It has been established that optic nerves transected in larval zebrafish are prone to misguided axon growth, including both contralateral and ipsilateral growth as well as only ipsilateral growth [34]. It was reported that approximately 50% of larvae displayed improper trajectories toward the optic tecta following transection [34], which was also seen here (Fig 6). Quantification of the appearance of normal regeneration versus formation of primarily a nerve stump revealed that fish expressing induced DN-Jun(+) were nearly three times more likely than controls to fail to regenerate damaged axons (Fig 6F, percentage of Red). Axon regeneration between DN-Jun(-) and DN-Jun(+) larvae, as it was categorized here based only on visible GFP axon projections, showed no significant difference in contralateral, contralateral and ipsilateral, or ipsilateral regrowth (Fig 6). Therefore, it is unlikely that Jun is necessary for axon guidance in this context. However, the occurrence of nerve stumps was significantly higher in DN-Jun(+) fish compared to DN-Jun(-); despite RGCs appearing to survive based on the presence of GFP. Therefore, it can be concluded that Jun is a key factor for RGC axon regeneration after transection.

It was demonstrated that larvae expressing DN-Jun were less likely to regenerate damaged RGC axons. Understanding of the regulatory chain of events necessary for successful optic nerve regeneration is critical; therefore, Jun putative target genes were next examined to determine whether their expression was impacted by the induction of DN-Jun. Target genes were selected based on their peak expression values previously identified in adult zebrafish optic nerve regeneration [20]. The selected genes are known transcription factors that could sustain the temporal cascades of gene expression during RGC axon regeneration. Because these genes had demonstrated clear differences in expression during adult regeneration, it was hypothesized that they would display similar expression patterns during larval optic nerve regeneration at distinct time points. Targets that demonstrated a large difference in log fold change of expression during adult zebrafish optic nerve regeneration [20] and associated with CNS and PNS regeneration were selected for further analysis. The selected Jun putative transcription factor targets included: *klf7b, sox11a, stat5a, ascl1a, e2f8, atf3, and nifl3.Klf7b, Sox11a* and *Stat5a* are known to function during neuronal development [56–59]. *Ascl1a* has been shown to promote neuronal differentiation in a variety of cell types including cortical neurons, glioblastoma stem cells, and mouse embryonic stem cells [60–62]. *E2f8* is a transcription factor involved with both cell proliferation and apoptosis [63], and its upregulation is associated with differentiation of neuronal stem cells [64]. Regarding the CNS, *Atf3, Ascl1a, Klf7,* and *Sox11a* have been shown to promote axon regeneration after injury [65–68]. Finally, in the context of the PNS, *Nfil3* was demonstrated to be a repressor that, when downregulated, enhances axon growth due to its repression of *CREB* and *C/EBP* [69,70].

Putative target gene expression was measured via RT-qPCR at each time point of regeneration. RNA was isolated from 30 dissected and pooled retinas at the time points presented for three independent experiments. First, to determine whether putative target expression levels in uninured controls were similar to uninjured adult expression values, putative target gene expression was compared between 0 hpt larval retinas and 0 dpi adult retinas (S2 Fig). Fold change was nearly identical for all genes with the exception of *ascl1a,* which was undetectable in adult retinas. This could be due to its function as a pioneer factor and role in differentiation of neural progenitor cells [62,71–73].

The average fold change in gene expression was plotted from the three individual replicates for both *Tg(isl2b:GFP)* X *Tg(isl2b:GFP)* progeny and *Tg(isl2b:GFP) X Tg(mke15Tg)*, DN-Jun(+) progeny (Fig 7). *Tg(isl2b:GFP)* X *Tg(isl2b:GFP)* progeny were used as controls for these experiments based on results from Fig 4 and Table 2 that indicated potential DN-Jun expression without detectable mCherry in DN-Jun(-) larvae. In *Tg(isl2b:GFP)* larvae, three putative targets, *atf3, ascl1a* and *sox11a* (along with *jun*) were significantly upregulated after optic nerve injury (Fig 7A–D, I and S3 Table). Genes that did not appear to have a significant change in expression after transection were *nfil3, klf7b, e2f8,* and the *stat5a-202* transcript (Fig 7E–I and S3 Table).

Variations in expression patterns were observed in DN-Jun(+) retinas over the regeneration timeline. *atf3* was significantly downregulated over the entire course of regeneration (Fig 7B, I), and *ascl1a* displayed discernable downregulation from 24 to72 hpt (age: 6 – 8 dpf) (Fig 7C, I). Some targets including *nfil3, klf7b*, *e2f8,* and *stat5a* had no significant change in expression (orange lines, Fig 7E–H), similar results were found when detecting multiple transcripts for *e2f8* and *stat5a* (S4 Fig). However, *sox11a* was upregulated with DN-Jun expression (Fig 7D, I). These patterns of upregulation or downregulation can be observed collectively to distinguish change in putative targets’ expression (Fig 7I). *atf3* remains significantly impacted by Jun knockdown during the regeneration time course. Its association with CNS regeneration [68] aligns with the increased likelihood of a nerve stump in DN-Jun(+) larvae (Fig 6E–F). It is possible that *klf7b, nfil3*, *stat5a-202,* and *e2f8* are not direct targets of Jun at this time, and that *sox11a* could be repressed by Jun. These varied trends suggest that Jun may act in a role other than a transcriptional activator, or that another intermediate factor could modulate the effect on these putative targets’ expression in this larval optic nerve transection model for regeneration.

## Discussion

*Jun* has long been associated with regeneration across the PNS and CNS in mammals and non-mammalian vertebrates [20,22–24,74,75]. Despite this, its necessity for functional regeneration in the CNS of non-mammalian vertebrates capable of functional regeneration has not been described. Furthermore, targets of Jun’s transcriptional activity remain to be fully understood in this context. Here, a recently developed larval zebrafish model of optic nerve regeneration [34], combined with the development of a heat-shock inducible functional Jun knockdown [27], were used to determine the necessity of Jun during successful larval optic nerve regeneration and its potential role in regulation of regeneration associated genes (RAGs).

It was established that upregulation of *jun* expression in the larval retina was a consequence of RGC axon injury, separate from developmentally expressed *jun* (Figs 1 and 3). This pattern of early induction is consistent in mouse, rat, hamster, and adult zebrafish optic nerve injury models [76–80]. These findings led to the hypothesis that early upregulation of *jun* following axonal injury may be necessary to initiate RAG expression. Jun has been implicated as an early response gene in a variety of regeneration models [20,81,82]. For example, in rat lesioned rubrospinal neurons and rat RGCs it has been demonstrated that Jun is strongly expressed after injury, which will poise the cells for regeneration amidst a high risk for apoptosis [74]. *jun* has also been found to promote axon growth in cultured and injured rat cortical neurons [82] and is upregulated in surviving, regenerating hamster RGCs [77]. Jun knockout in mammalian optic nerve injury models resulted in diminished axon regrowth [79,80].

In contrast, and despite the known necessity for *jun* within the regenerating PNS [23,24,83], it has been noted that Jun function can differ between the PNS and CNS [81,82] and is commonly associated with apoptosis after injury in some mammalian models [80,84–89], and in developing retinal neurons [90]. It has also been shown that that low levels of Jun are associated with cell survival and failure to regenerate, resulting in a nerve stump [74]. Therefore, it is possible that the extent of Jun upregulation could determine RGC fate after injury and that the exact role of Jun in regeneration may vary depending on the system, cell type, and extent of Jun upregulation.

In these studies, the role of Jun during development was differentiated from its role in axon regeneration by manipulation of the timing for DN-Jun induction. DN-Jun induction during embryonic development resulted in anterior defects of larval zebrafish, most notably underdeveloped jaw cartilage and diminished growth of cranial structures (Fig 4), whereas optic nerve transection in larval zebrafish with RGCs expressing DN-Jun failed to regenerate (Fig 6). This is the first evidence demonstrating the importance of Jun function during optic nerve regeneration in a system capable of functional CNS regeneration. Important for these results is the finding that heat shock inducible DN-Jun acted by both outcompeting endogenous Jun, and subsequently preventing *jun* upregulation during larval zebrafish optic nerve regeneration (Figs 4 and 5). It fact, it has been shown that *Jun* is capable of autoregulation in a variety of mammalian systems [55,85,91] and it was suggested in previous work that *jun* was capable of autoregulation in zebrafish regeneration but had not been functionally tested [20]. It is important to note that DN-Jun expression was unable to be directly quantified as it contains the sequence for the endogenous *jun* DNA binding domain*.* Although mCherry was able to be visualized 3 days after heat shock, this was not a direct indication of the presence or amount of DN-Jun.

It has been suggested that Jun’s additional role is to function as a core regulator of downstream regeneration-associated transcription factor expression during axon regeneration [83,92,93]. To further expand upon the importance of Jun during optic nerve regeneration in zebrafish, the expression of select putative Jun target genes previously identified in Dhara et. al., 2019 were evaluated (Fig 7). Putative targets displayed different trends in expression during normal larval optic nerve regeneration. The upregulation of *atf3*, *ascl1a,* and *sox11a* was consistent with the previous studies demonstrating their upregulation after neuronal injury [20,66,67,78,93–96]. However, the expression profiles identified for *klf7b, nfil3, e2f8,* and *stat5a-202* were unexpected given their known upregulation in adult zebrafish optic nerve regeneration and PNS regeneration [20,69,97,98].

Putative target expression was further measured in retinas expressing DN-Jun during the regeneration time course. The effects of DN-Jun on some putative targets align with the increased likelihood of a nerve stump after transection. Most notably, a*tf3* was significantly downregulated with functional Jun knockdown (Fig 7B,I), indicating that it is likely a target of Jun after optic nerve injury. These results are consistent with previous findings showing *Atf3* to be necessary for survival in injured neurons [96,99] and that ATF3 can be regulated by c-Jun in rat cerebellar granule neurons [100]. Further, Jun:Atf3 dimer overexpression was demonstrated to promote neurite outgrowth in cultured rat dorsal root ganglia (DRG), cortical, and hippocampal neurons [24,75]. Together these findings suggest a critical role for Jun regulation of ATF3 and their potential interaction in optic nerve regeneration. *Ascl1a* morpholino-mediated knockdown in adult zebrafish optic nerve regeneration was found to significantly reduce RGC axon growth [66]. However, downregulation of *ascl1a* in the context of Jun knockdown has not previously been shown, as no regulatory relationship between the two factors has been identified until now. This novel regulatory relationship implicating *ascl1a* as a functional target of Jun suggests that Jun acts earlier upon injury-induced signaling than previously thought to implement open chromatin for *ascl1a* upregulation. Additionally, there could be a discrepancy between *ascl1* gene expression within RGCs and Müller Glia, as whole retinas were used in these experiments and *Ascl1a* is known to be upregulated within Müller Glia after optic nerve injury [101]. Upregulation of *sox11a* aligns with previous findings in mammalian and zebrafish optic nerve injury [20,78,102]. This indicates a consistent role in RGC survival and possible axon outgrowth.

Although Jun activity is typically associated with transcriptional activation, significant upregulation was observed for *sox11a* at select time points with Jun knockdown during regeneration. This novel relationship requires further investigation to determine either direct or indirect regulation via Jun, and whether this response is localized to RGCs. An additional consideration would be the role of Jun in targeting alternate transcripts for these putative target genes. For example, the *stat5a-202* transcript was shown to be upregulated during adult zebrafish optic nerve regeneration [20] and was hypothesized to show a similar pattern in larvae. However, the *stat5a-202* transcript showed a trend of downregulation after injury in control larvae and its overall expression increased in the presence of DN-Jun after injury. Therefore, it is possible that Jun may have a broader role as a repressor for transcript variants of RAGs as well, and the effects of its activity should be further investigated.

Finally, *nfil3* which acts as a repressor for RAG expression in axotomized peripheral DRG neurons [69] here, had no significant change in expression in either control or DN-Jun conditions. Therefore, *nfil3* is not likely to be necessary for larval RGC axon regeneration, but it could have a role in fine tuning regulation in regenerating adult RGCs. The absence of significant *klf7b* upregulation was surprising as well, given its frequent upregulation upon injury and association with axonal outgrowth in the CNS [65,78,103].

In trying to elucidate Jun target genes during optic nerve regeneration, it is important to consider the potential differences in studying a zebrafish larval versus adult regeneration system. While it has been established that the zebrafish visual system is mature and functional by 5 dpf [34,49,51,104] the larval optic nerve may not be fully analogous to the adults with important differences to consider in regards to myelination and regulation of axon guidance. Myelination is first detected within the optic chiasm at 2 days post fertilization in zebrafish and continues to develop across the optic nerve through 10 days post fertilization [105,106]; therefore, it is still developing during these regeneration studies which may influence RAG expression upon injury.

It is also important to consider gene regulation of axon guidance versus regeneration. Axon misguidance was demonstrated here (Fig 6) and has been described in larval zebrafish optic nerve regeneration [34]. The same misguidance in adult zebrafish optic nerve regeneration was not examined by Dhara et al 2019; although previous studies in adult fish have reported fully contralateral regeneration [107], considerable ipsilateral regeneration in goldfish [108,109], and misguided axons in up to 59% of injured animals [109]. Here, retinal dissection was conducted on all transected and regenerating retinas regardless of axon regeneration guidance defects. Therefore, the population of regenerating RGCs that were examined is heterogeneous which could have obscured RAG expression levels during regeneration. Further studies distinguishing RAG expression, and the influence of DN-Jun on RAG expression, between full contralateral regeneration, ipsilateral regrowth, and nerve stump outcomes could provide further insight into genes regulated during regeneration and by Jun. Since the DN-Jun expression in this model is ubiquitous in the embryo, the possibility that surrounding cells or tissues near the RGCs and their axons are also playing a Jun-mediated role in regeneration cannot be ruled out. An RGC specific DN-Jun model would be required to determine true cell specificity.

Another variable in this model that could have influenced RAG expression is the heat shock used to induce DN-Jun. Heat shock proteins (HSPs) are rapidly upregulated in response to axonal injury and shown to be involved with axonal transport and neuroprotection [110–112]. Patients with glaucoma that lack intraocular pressure have elevated levels of anti-HSP antibodies in their sera as well [113]. Because HSPs are neuroprotective and stabilize the cytoskeleton and optic nerve head [114], our experimental heat shock induction model could influence cell survival and subsequently capacity for regeneration. Future studies would benefit from distinguishing between the neuroprotective effects of HSPs and the potential apoptotic role of Jun on RGC survival. Although, zebrafish optic nerve regeneration is specific to the injured RGCs, and independent of new progenitor axon growth [16,34]. Moreover, the regulation of Jun’s putative target genes could be directly or indirectly influenced by heat shock, and further understanding of whether Jun directly regulates these targets is required. Finally, complete axon transection in the larval model compared to the adult optic nerve crush could result in different RAG expression, differential RGC survival, and differences in the regeneration progress [20,115].

Despite these limitations, the heat shock inducible DN-Jun transgenic line has proven to be a valuable zebrafish model to study the importance of Jun not only in optic nerve regeneration, but regeneration of other CNS and non-neuronal tissues. Jun is known to be an early injury-induced response gene, upregulated proportionally to the extent of damage in traumatic injury and glaucoma models [88] and inactivation associated with RGC survival after injury [86]. Here, evidence that a loss of Jun results in an increased likelihood for a nerve stump, despite its association with apoptosis, is important for RGC axon regeneration. It is likely that fine-tuned regulation of *jun* will be necessary to further understand the regulatory programming for successful optic nerve regeneration. Furthermore, this study demonstrates the varying patterns of RAG expression after injury and can further define the key transcription factor core regulators necessary for axon regeneration. Therefore, the highly conserved Jun protein remains an attractive target for potential therapies to treat CNS injury and neurodegenerative diseases.

## Supporting information

S1 Table *Tg(hsp70l:dnjun-2A-mCherry,Cryoaa:EGFP)* zebrafish line generation. The number of animals used for each step is outlined, resulting in 3 founder lines used for these studies.
(DOCX)

S2 Table Ensembl transcript IDs for all genes used for qPCR experiments. Ensembl GRCz11.
(DOCX)

S3 Table Comparison of fold change in gene expression significance in *Tg(isl2b:GFP)* larvae. All transcripts and primers for these data are shown in Table 1. Arrows (when indicated) signify an expression trend of upregulation (up arrow, **⇧**) or downregulation (down arrow, **⇩**). One-way ANOVA with Tukey’s Multiple Comparison post-test was used to determine significance of gene expression between timepoints. *  =  p < 0.05, ** =  p < 0.01, *** =  p < 0.001, **** =  p < 0.0001.
(DOCX)

S1 Fig glmp expression is consistent during optic nerve regeneration in Tg(isl2b:GFP) larvae. (**A-C**) Amplification plots of individual replicates of *glmp* at 0 hpt, 6 hpt, 24 hpt, 48 hpt, 96 hpt, and 120 hpt in *Tg(isl2b:GFP)* larvae.
(DOCX)

S2 Fig Jun putative target expression is consistent between larval and adult retinas with the exception of *ascl1a.
* The average of three technical replicates of 6 pooled, 0 dpi adult zebrafish retinas, and 30 pooled, 0 hpt larval retinas were used for RT-qPCR analyses. Error bars represent standard deviation.
(DOCX)

S3 Fig Individual replicates of Jun putative target gene expression in *Tg(isl2b:GFP)* and DN-Jun(+) Fish. Plotted averages are found in Fig 7.
(DOCX)

S4 Fig Average expression of multiple *e2f8* and *stat5a* transcripts in *Tg(isl2b:GFP)* and DN-Jun(+) fish. RT-qPCR of putative Jun target genes’ showing average expression during regeneration in *Tg(isl2b:GFP)* X *Tg(isl2b:GFP),* (green) and Jun knockdown *Tg(isl2b:GFP)* X *Tg(mke15Tg)* DN-Jun(+), (orange) fish. (**A–B**) Average fold change of three individual replicates are plotted for each condition and timepoint. Each timepoint required n =  90 retinas (30 retinas per replicate for 3 replicates) for both control and knockdown experiments. *e2f8* forward 5’-TCTTCGTGAAACCCATGTCA-3’ *e2f8* reverse 5’-GACCGCCTTTAGGTGTGGTA-3’; *stat5a* forward 5’-TGACCCGAGAAGCTAACACC-3’ *stat5a* reverse 5’-GTATGTCCAGTCCTCCCT-3’. (**C–E**) Individual replicates for averages shown in A and B. *Tg(isl2b:GFP)* are shown in C and D. DN-Jun(+) are shown in E and F.
(DOCX)
