# Supplementary material for: The transcription factor Jun is necessary for optic nerve regeneration in larval zebrafish
Source: PLoS One. 2025 Mar 10;20(3):e0313534. doi: 10.1371/journal.pone.0313534 (PMC11892826; doi:10.1371/journal.pone.0313534)
Supplement: S1 Table — The number of animals used for each step is outlined, resulting in 3 founder lines used for these studies. (DOCX) [file pone.0313534.s001.docx]

**S1 Table. *Tg(hsp70l:dnjun-2A-mCherry,Cryoaa:EGFP)*** **zebrafish line generation.**

| *Tg(hsp70l:dnjun-2A-mCherry,Cryoaa:EGFP)*  **Zebrafish Line Generation** | |
| --- | --- |
| **Step** | **Number of Animals Used** |
| Eggs Injected | 1730 |
| Survivors | 567 |
| F0 Cryoaa:EGFP+ | 114 (20.1%) |
| F0 Germline Transmission | 6 out of 84 (7.1%) |
| F1 HS- Cryoaa:EGFP+, mCherry- | 3 |

The number of animals used for each step is outlined, resulting in 3 founder lines used for these studies.
