# Supplementary material for: The transcription factor Jun is necessary for optic nerve regeneration in larval zebrafish
Source: PLoS One. 2025 Mar 10;20(3):e0313534. doi: 10.1371/journal.pone.0313534 (PMC11892826; doi:10.1371/journal.pone.0313534)
Supplement: S2 Table — Ensembl GRCz11. (DOCX) [file pone.0313534.s002.docx]

**S2 Table. Ensembl transcript IDs for all genes used for qPCR experiments.**

| **Transcript Name** | **ENSDART** |
| --- | --- |
| ascl1a | ENSDART00000056005 |
| atf3 | ENSDART00000022060 |
| e2f8 | ENSDART00000128488 |
| jun | ENSDART00000063912 |
| insm1a | ENSDART00000124040 |
| klf7b | ENSDART00000104317 |
| nfil3 | ENSDART00000138821 |
| stat5a | ENSDART00000139763 |

Ensembl GRCz11.
