# Supplementary material for: The transcription factor Jun is necessary for optic nerve regeneration in larval zebrafish
Source: PLoS One. 2025 Mar 10;20(3):e0313534. doi: 10.1371/journal.pone.0313534 (PMC11892826; doi:10.1371/journal.pone.0313534)
Supplement: S3 Table — All transcripts and primers for these data are shown in Table 1. Arrows (when indicated) signify an expression trend of upregulation (up arrow, ⇧) or downregulation (down arrow, ⇩). One-way ANOVA with Tukey’s Multiple Comparison post-test was used to determine significance of gene expression between timepoints. * = p < 0.05, ** = p < 0.01, *** = p < 0.001, **** = p < 0.0001. (DOCX) [file pone.0313534.s003.docx]

**S3 Table. Comparison of fold change in gene expression significance in *Tg(isl2b:GFP)*.**

| Tukey’s Multiple Comparison of Fold Change in Gene Expression Significance in *Tg(isl2b:GFP)* | | | | | | | |
| --- | --- | --- | --- | --- | --- | --- | --- |
|  | ***atf3***  **⇧** | ***ascl1***  **⇧** | ***klf7b*** | ***sox11***  **⇧** | ***stat5a*** | ***e2f8***  **⇩** | ***nfil3*** |
| 0 hpt vs 6 hpt |  |  |  |  |  |  |  |
| 0 hpt vs 24 hpt | *** |  |  | ** |  |  |  |
| 0 hpt vs 48 hpt | ** |  |  |  |  | ** |  |
| 0 hpt vs 72 hpt |  |  |  |  |  | *** |  |
| 0 hpt vs 96 hpt |  |  |  |  |  | *** |  |
| 0 hpt vs 120 hpt |  |  |  |  |  | *** |  |
| 6 hpt vs 24 hpt | *** |  |  | ** |  |  |  |
| 6 hpt vs 48 hpt |  |  |  |  |  |  |  |
| 6 hpt vs 72 hpt |  |  |  |  |  |  |  |
| 6 hpt vs 96 hpt |  |  |  |  |  | ** |  |
| 6 hpt vs 120 hpt |  |  |  |  |  | ** |  |
| 24 hpt vs 48 hpt | *** |  |  |  |  |  |  |
| 24 hpt vs 72 hpt | *** |  |  | ** |  |  |  |
| 24 hpt vs 96 hpt | *** | * |  | *** |  | * |  |
| 24 hpt vs 120 hpt | *** | * |  | *** |  | * |  |
| 48 hpt vs 72 hpt |  |  |  |  |  |  |  |
| 48 hpt vs 96 hpt |  | * |  | * |  |  |  |
| 48 hpt vs 120 hpt |  | * |  | ** |  |  |  |
| 72 hpt vs 96 hpt |  |  |  |  |  |  |  |
| 72 hpt vs 120 hpt |  |  |  |  |  |  |  |
| 96 hpt vs 120 hpt |  |  |  |  |  |  |  |

All transcripts and primers for these data are shown in Table 1. Arrows (when indicated) signify an expression trend of upregulation (up arrow, **⇧**) or downregulation (down arrow, **⇩**). One-way ANOVA with Tukey’s Multiple Comparison post-test was used to determine significance of gene expression between timepoints. * = p<0.05, ** = p<0.01, *** = p<0.001, **** = p<0.0001.
