# Supplementary material for: The transcription factor Jun is necessary for optic nerve regeneration in larval zebrafish
Source: PLoS One. 2025 Mar 10;20(3):e0313534. doi: 10.1371/journal.pone.0313534 (PMC11892826; doi:10.1371/journal.pone.0313534)
Supplement: S1 Fig — (A-C) Amplification plots of individual replicates of glmp at 0 hpt, 6 hpt, 24 hpt, 48 hpt, 96 hpt, and 120 hpt in Tg(isl2b:GFP) larvae. (DOCX) [file pone.0313534.s004.docx]

**S1 Fig. *glmp* expression is consistent during optic nerve regeneration in *Tg(isl2b:GFP)* larvae.**


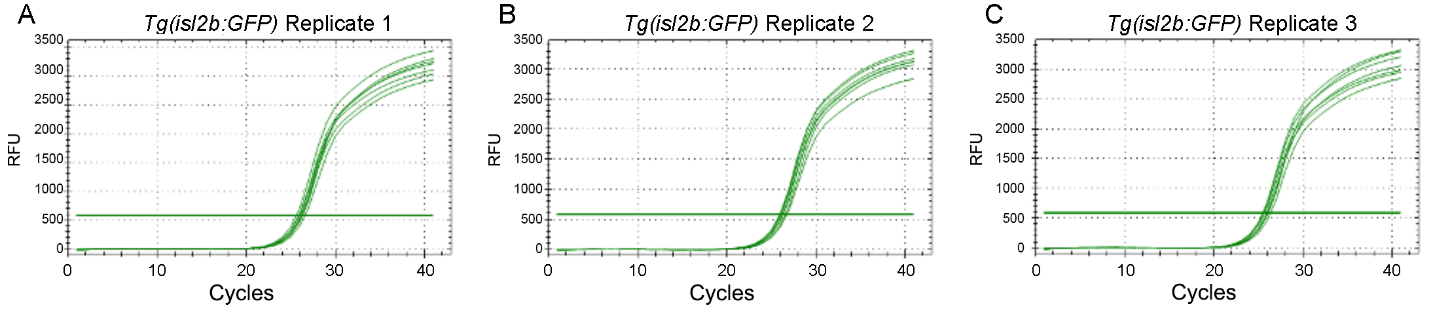


(**A-C**) Amplification plots of individual replicates of *glmp* at 0 hpt, 6 hpt, 24 hpt, 48 hpt, 96 hpt, and 120 hpt in *Tg(isl2b:GFP)* larvae.
