# Supplementary material for: The transcription factor Jun is necessary for optic nerve regeneration in larval zebrafish
Source: PLoS One. 2025 Mar 10;20(3):e0313534. doi: 10.1371/journal.pone.0313534 (PMC11892826; doi:10.1371/journal.pone.0313534)
Supplement: S2 Fig — The average of three technical replicates of 6 pooled, 0 dpi adult zebrafish retinas, and 30 pooled, 0 hpt larval retinas were used for RT-qPCR analyses. Error bars represent standard deviation. (DOCX) [file pone.0313534.s005.docx]

**S2 Fig. Jun putative target expression is consistent between larval and adult retinas with the exception of *ascl1a*.**


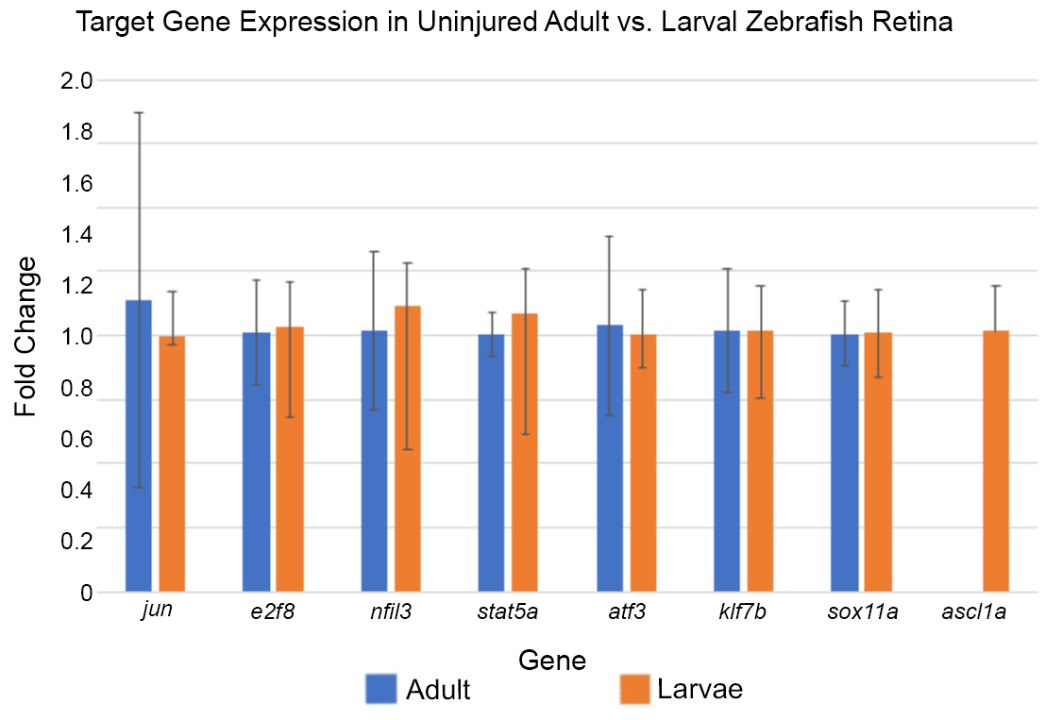


The average of three technical replicates of 6 pooled, 0 dpi adult zebrafish retinas, and 30 pooled, 0 hpt larval retinas were used for RT-qPCR analyses. Error bars represent standard deviation.
