# Supplementary material for: The transcription factor Jun is necessary for optic nerve regeneration in larval zebrafish
Source: PLoS One. 2025 Mar 10;20(3):e0313534. doi: 10.1371/journal.pone.0313534 (PMC11892826; doi:10.1371/journal.pone.0313534)
Supplement: S3 Fig — Plotted averages are found in Fig 7. (DOCX) [file pone.0313534.s006.docx]

**S3 Fig. Individual replicates of Jun putative target gene expression in *Tg(isl2b:GFP)* and DN-Jun(+) fish.**


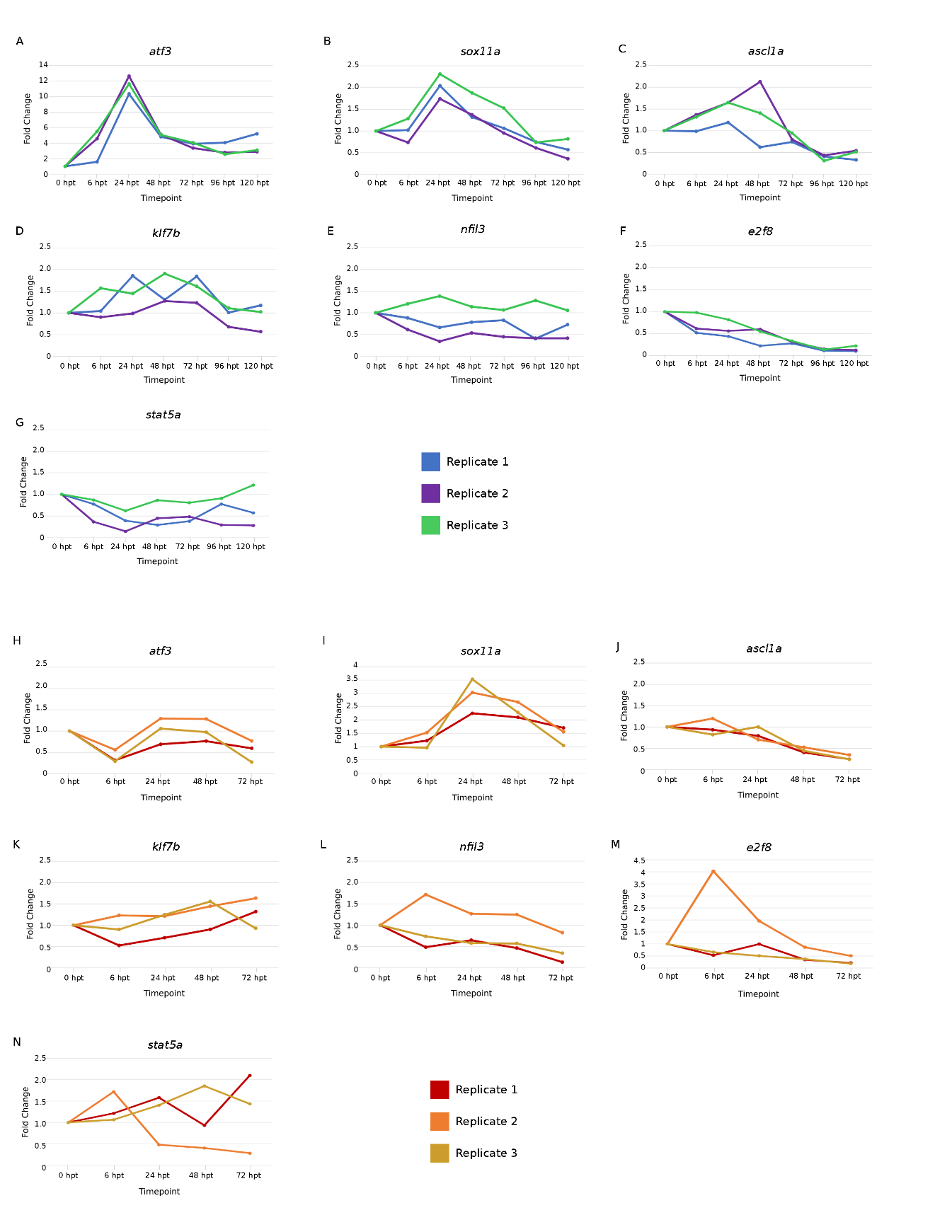


Plotted averages are found in Figure 7.
