# Supplementary material for: The transcription factor Jun is necessary for optic nerve regeneration in larval zebrafish
Source: PLoS One. 2025 Mar 10;20(3):e0313534. doi: 10.1371/journal.pone.0313534 (PMC11892826; doi:10.1371/journal.pone.0313534)
Supplement: S4 Fig — RT-qPCR of putative Jun target genes’ showing average expression during regeneration in Tg(isl2b:GFP) X Tg(isl2b:GFP), (green) and Jun knockdown Tg(isl2b:GFP) X Tg(mke15Tg) DN-Jun(+), (orange) fish. (A–B) Average fold change of three individual replicates are plotted for each condition and timepoint. Each timepoint required n = 90 retinas (30 retinas per replicate for 3 replicates) for both control and knockdown experiments. e2f8 forward 5’-TCTTCGTGAAACCCATGTCA-3’ e2f8 reverse 5’-GACCGCCTTTAGGTGTGGTA-3’; stat5a forward 5’-TGACCCGAGAAGCTAACACC-3’ stat5a reverse 5’-GTATGTCCAGTCCTCCCT-3’. (C–E) Individual replicates for averages shown in A and B. Tg(isl2b:GFP) are shown in C and D. DN-Jun(+) are shown in E and F. (DOCX) [file pone.0313534.s007.docx]

**S4 Fig. Average expression of multiple *e2f8* and *stat5a* transcripts in *Tg(isl2b:GFP)* and DN-Jun(+) fish.**


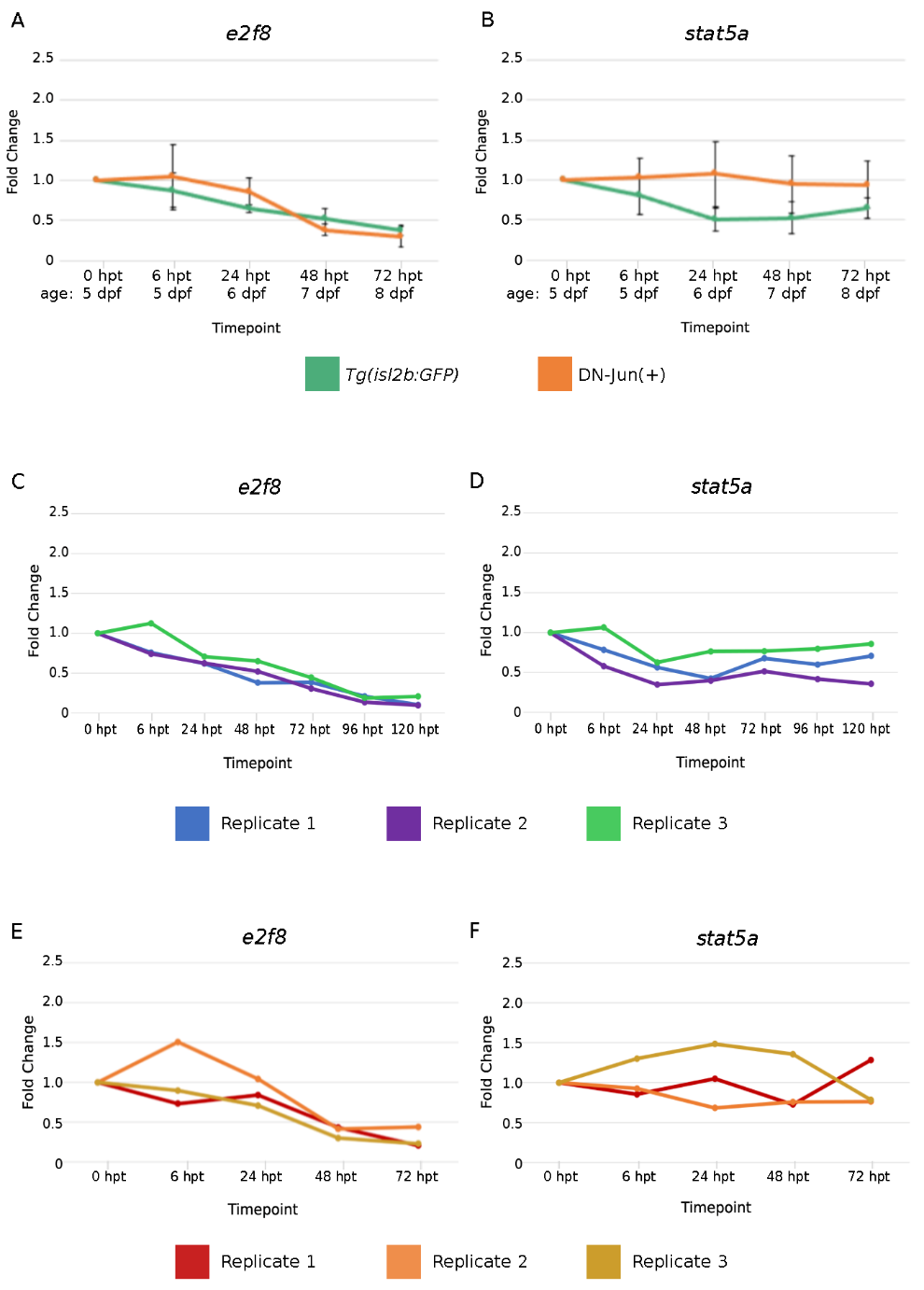


RT-qPCR of putative Jun target genes’ showing average expression during regeneration in *Tg(isl2b:GFP)* X *Tg(isl2b:GFP),* (green) and Jun knockdown *Tg(isl2b:GFP)* X *(mke15Tg)* DN-Jun(+), (orange) fish. (**A-B**) Average fold change of three individual replicates are plotted for each condition and timepoint. Each timepoint required n = 90 retinas (30 retinas per replicate for 3 replicates) for both control and knockdown experiments. *e2f8* forward 5’-TCTTCGTGAAACCCATGTCA-3’ *e2f8* reverse 5’-GACCGCCTTTAGGTGTGGTA-3’ [1]; *stat5a* forward 5’-TGACCCGAGAAGCTAACACC-3’ *stat5a* reverse 5’-GTATGTCCAGTCCTCCCT-3’ [2]. (**C-E**) Individual replicates for averages shown in A and B. *Tg(isl2b:GFP)* are shown in C and D. DN-Jun(+) are shown in E and F.

**References:**

1. Shimada Y, Kuninaga S, Ariyoshi M, Zhang B, Shiina Y, Takahashi Y, et al. E2F8 promotes hepatic steatosis through FABP3 expression in diet-induced obesity in zebrafish. Nutr Metab (Lond). 2015;12:17.
2. Peron M, Dinarello A, Meneghetti G, Martorano L, Facchinello N, Vettori A, et al. The stem-like Stat3-responsive cells of zebrafish intestine are Wnt/beta-catenin dependent. Development. 2020;147(12).
